# The role of vitamin A and zinc supplementation in pediatric pneumonia: A protocol for systematic review and network meta-analysis

**DOI:** 10.1097/MD.0000000000032183

**Published:** 2022-12-23

**Authors:** Yu Shi, Qihong Lei, Hongxia Liu, Qing Han

**Affiliations:** a Respiratory department, Children’s Hospital Affiliated to Nanjing Medical University, Nanjing, Jiangsu, China.

**Keywords:** network meta-analysis, pediatric pneumonia, protocol, vitamin A, zinc

## Abstract

There is still controversy regarding the role of zinc and vitamin A supplementation in pediatric pneumonia. Therefore, we designed a systematic review and network meta-analysis to incorporate the available evidence to determine the role of vitamin A and zinc in pediatric pneumonia.

**Methods:**

From the inception to December 31, 2022, the Web of Science, EMBASE, Medicine, the Cochrane Central Register of Controlled Trials and Cochrane Library electronic databases were searched using the key phrases “pneumonia,” “pediatric,” “zinc,” “vitamin A,” and “cohort” for all relevant studies. Cohort studies assessing the efficacy and safety of zinc and/or vitamin A supplementation in children with pneumonia will be included for quantitative or qualitative analysis. Non-cohort studies and studies that do not meet the inclusion criteria will be excluded. Outcome measures include reduction in duration of pneumonia, adverse drug events, length of hospital stay, amount of antibiotics used, and radiological parameters. The primary outcome is the degree of reduction in the duration of pneumonia.

**Results:**

This Bayesian network meta-analysis will be conducted in accordance with the PRISMA extension statement, which is used to report systematic reviews that incorporated a meta-analysis of medical intervention networks.

## 1. Introduction

Pneumonia in children is caused by viral or bacterial pathogens. Worldwide, pneumonia is the leading cause of pediatric morbidity and mortality. It is estimated that more than 2 million children under 5 years of age die from pneumonia each year, accounting for 20% of the annual deaths in this age group.^[1–3]^ Treatment of pneumonia includes antimicrobial drugs, supportive management with supplemental oxygen, intravenous fluids, and antipyretics. Immunization, breastfeeding, adequate nutrition and good hygiene can help prevent pneumonia.^[4]^

Vitamin A plays an important pleiotropic role in supporting a normal mucosal barrier. Vitamin A deficiency has been found to increase the risk of invasive pathogens by causing squamous respiratory epithelial metaplasia, impaired airway mucosal barrier, delayed repair, and decreased airway antioxidants.^[5–7]^ Children with vitamin A deficiency are more likely to develop acute respiratory infections and diarrhea. Zinc is essential for gene expression and plays an important role in nucleic acid and protein synthesis, cell replication, tissue growth and repair.^[8,9]^ Therefore, zinc plays an important role in the development and maintenance of host defense against infection. The therapeutic benefit of oral zinc in diarrhea has been well documented in a review of 18 trials by Cochrane, which showed that oral zinc shortened the recovery time of children with acute or persistent diarrhea in the 6 months to 5 years age group. It has also been shown that routine zinc supplementation reduces the risk of acute respiratory tract infections and clinical pneumonia in children.^[10]^

There is still controversy regarding the role of zinc and vitamin A supplementation in pediatric pneumonia. More specifically, the literature remains unclear regarding the advantages and disadvantages of zinc supplementation alone, vitamin A supplementation alone, and combined zinc and vitamin A supplementation. No comprehensive review has attempted to address this issue; therefore, we designed a systematic review and network meta-analysis to incorporate the available evidence to determine the role of vitamin A and zinc in pediatric pneumonia.

## 2. Materials and methods

### 2.1. Search strategy

From the inception to December 31, 2022, the Web of Science, EMBASE, Medicine, the Cochrane Central Register of Controlled Trials and Cochrane Library electronic databases were searched using the key phrases “pneumonia,” “pediatric,” “zinc,” “vitamin A,” and “cohort” for all relevant studies. Moreover, references cited by the relevant sources will also be hand-searched to identify any additional articles that can not be found in our database query.

### 2.2. Study registration and ethical consideration

The protocol was registered on PROSPERO, with the registration number CRD42022371391. We will update our protocol for any changes in the entire research process if needed. Given that the study data are based on a database study, ethical approval does not seem to apply to this paper.

### 2.3. Eligibility criteria

This Bayesian network meta-analysis will be conducted in accordance with the PRISMA extension statement, which is used to report systematic reviews that incorporated a meta-analysis of medical intervention networks. Cohort studies assessing the efficacy and safety of zinc and/or vitamin A supplementation in children with pneumonia will be included for quantitative or qualitative analysis. Non-cohort studies and studies that do not meet the inclusion criteria will be excluded. In addition, there are no language and publication time restrictions for this study. The following PICO protocol is drafted to guide the search:

P (population): Pediatric patients with pneumonia

I (intervention): Group with vitamin A plus zinc

C (comparison): Group with vitamin A or group with zinc or placebo

O (outcomes): Outcome measures include reduction in duration of pneumonia, adverse drug events, length of hospital stay, amount of antibiotics used, and radiological parameters. The primary outcome is the degree of reduction in the duration of pneumonia.

### 2.4. Selection of studies and data extraction

Each trial will be independently assessed by 2 reviewers for screening and data extraction. In case of disagreement, consensus is reached through discussion among reviewers or consultation with senior scholars. We will screen for trial eligibility, extract data, and reach consensus using a standardized and piloted web-based data management tool and a codebook. We will extract trial features such as design, size, and duration; characteristics of the intervention, such as dose and duration of treatment; participant characteristics such as mean age, sex, Body Mass Index, and mean duration of symptoms; as well as the outcome data for each time point of interest. Where necessary, we approximate summary statistics and variability measures from the charts. Where possible, outcomes based on intention-to-treat principles are extracted.

### 2.5. Statistical analysis

The network meta-analyses will be performed using STATA/MP 14.1 (StataCorp, College Station, TX), through the routine for Bayesian hierarchical random-effects model. The network meta-analysis combines data from several different randomized comparisons across treatments to provide a set of internally consistent estimates, taking into account randomization within each trial. The network meta-analysis will be performed within the framework of a generalized linear model, where the link function specifies the relationship between the outcome and the model coefficients to be estimated. When the endings are continuous, the likelihood is modeled as normal. When the endings are event rates, the likelihood is modeled as Poisson. A random effects model is used for this analysis. Estimation is performed in a Bayesian context using the non-information prior distribution of the parameters. The model is evaluated using the Deviation Information Criterion, a measure that combines model fit and complexity. The analysis is estimated using a Bayesian Markov Chain Monte Carlo model.

### 2.6. Risk of bias and quality assessments

Two investigators will separately assess the methodological quality of the included studies. A modified Oxford score (ranging from 0–7) is used to measure the methodological quality of all studies as described in the randomization, masked assignment, blinding, and participant withdrawal reports.^[11]^ Any controversy will be resolved by discussing with a third author to reach a final consensus.

### 2.7. Sensitivity analysis and subgroup analysis

Study quality will be assessed using a modified Cochrane risk of bias assessment tool. Sensitivity analyses are performed on the 2 items with the highest risk of bias and on the included studies. Subgroup analyses are performed to assess differences in the efficacy of different doses of zinc and vitamin A in patients.

## 3. Discussion

Zinc deficiency can lead to growth disorders, loss of appetite, behavioral changes and impaired immune function, resulting in susceptibility to infections. Regarding vitamin A, it is 1 of the most important micronutrients and is essential for many vital functions such as cell growth, epithelial integrity and immune system response. There is still controversy regarding the role of zinc and vitamin A supplementation in pediatric pneumonia. More specifically, the literature remains unclear regarding the advantages and disadvantages of zinc supplementation alone, vitamin A supplementation alone, and combined zinc and vitamin A supplementation. No comprehensive review has attempted to address this issue; therefore, we designed a systematic review and network meta-analysis to incorporate the available evidence to determine the role of vitamin A and zinc in pediatric pneumonia. We hypothesized that combined zinc and vitamin A supplementation would minimize the rate of healing and mortality of pediatric pneumonia.

## Author contributions

**Conceptualization:** Qing Han.

**Data curation:** Yu Shi, Qihong Lei.

**Formal analysis:** Yu Shi, Qihong Lei.

**Funding acquisition:** Qing Han.

**Investigation:** Yu Shi, Qihong Lei.

**Methodology:** Qing Han.

**Resources:** Qing Han.

**Supervision:** Hongxia Liu.

**Writing** – **original draft:** Yu Shi.

**Writing** – **review & editing:** Hongxia Liu.
